# Smelting Magnesium Metal using a Microwave Pidgeon Method

**DOI:** 10.1038/srep46512

**Published:** 2017-04-12

**Authors:** Yuji Wada, Satoshi Fujii, Eiichi Suzuki, Masato M. Maitani, Shuntaro Tsubaki, Satoshi Chonan, Miho Fukui, Naomi Inazu

**Affiliations:** 1 Department of Applied Chemistry, Graduate School of Science and Engineering, Tokyo Institute of Technology, 2-12-1Ookyama, Meguro-ku, Tokyo, 152-8550 Japan; 2 Oricon Energy Inc., 6-8-10 Roppongi, Minato-ku, Tokyo, 106-0032 Japan

## Abstract

Magnesium (Mg) is a lightweight metal with applications in transportation and sustainable battery technologies, but its current production through ore reduction using the conventional Pidgeon process emits large amounts of CO_2_ and particulate matter (PM2.5). In this work, a novel Pidgeon process driven by microwaves has been developed to produce Mg metal with less energy consumption and no direct CO_2_ emission. An antenna structure consisting of dolomite as the Mg source and a ferrosilicon antenna as the reducing material was used to confine microwave energy emitted from a magnetron installed in a microwave oven to produce a practical amount of pure Mg metal. This microwave Pidgeon process with an antenna configuration made it possible to produce Mg with an energy consumption of 58.6 GJ/t, corresponding to a 68.6% reduction when compared to the conventional method.

The low density and lightweight nature of magnesium (Mg) alloys make them desirable replacements for Al alloys in the automotive industry, as the subsequent reduction in the weight of automotive components results in an improvement in fuel economy. For example, a 100 kg reduction in the weight of a standard vehicle leads to a fuel saving of about 0.5 L per 100 km^1^,^2^. Mg also has the highest internal energy density for electricity, and as such, batteries using Mg as a negative electrode have a higher power storage capacity than Li ion batteries^3^. Based on this, Yabe proposed a fossil-fuel free energy cycle^4^,^5^, wherein the reaction product from Mg batteries is refined back to pure Mg using lasers. The lightweight nature of Mg, combined with its high energy density, suggests that the global consumption of this metal will increase drastically in the near future.

Currently, 85% of the world’s Mg metal is produced in China using the Pidgeon process, which was originally invented in Canada in the 1940s as a more economical alternative to the electrolytic process. In the Pidgeon process, crushed Mg ore (dolomite) is subjected to a calcination process (1,000–1,300 °C) that produces dolomite (CaO • MgO). The calcined product is subsequently crushed and mixed with ferrosilicon, an alloy of iron and silicon made by the reduction of silica sand with coke and iron scrap in an arc furnace at a temperature of 1,600 °C, and then formed into briquettes. These briquettes are fed into a batch vacuum reduction furnace operated at a high temperature of 1,160 °C and under a low pressure of 1–2 Pa to release Mg vapour. A water-cooled condenser is used to collect Mg as a pure solid (crown) before it is being re-melted and formed into ingots. The overall Mg production equation is as follows:


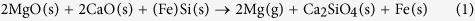


with (s) and (g) indicating the physical state of each chemical as solid or gas, respectively.

Approximately 10.4 kg of coal is burned to provide enough heat to obtain 1 kg of Mg, while simultaneously around 37 kg of CO_2_ gas is released^6^,^7^,^8^. Therefore, the impact of Mg metal production on climate change needs to be understood at a global scale and a new, energy-saving process for fabricating Mg ingots should be considered.

Chemical reactions performed under microwave irradiation often demonstrate high reaction rates and high selectivity, which allows for a more compact reactor and a more energy-efficient process than conventional heating. Microwave chemical processing and synthesis have therefore attracted significant attention as a means of improving process efficiency and conserving energy for realizing “green chemistry” and “green engineering”^9^,^10^,^11^,^12^. Some researchers have also proposed smelting metal using microwave heating in order to achieve a decrease in CO_2_ emissions^13^,^14^. In addition, Yoshikawa and Morita have reported the microwave-based carbothermic reduction of MgO, using amorphous carbon instead of ferrosilicon as the reduction agent, which yielded particles of Mg metal so tiny that they could only be identified by XRD measurement^14^.

In this study, we have investigated a new Pidgeon process, using microwaves instead of coal as the heat source, for producing Mg with lower energy inputs and a decrease in greenhouse gas emissions. Mg smelting was carried out using microwave irradiation with a multi-mode applicator and a magnetron source. The energy loss per unit volume caused by microwave irradiation can generally be given as:


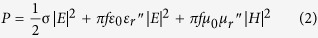


where, *P, E, H, σ, f, ε*_0_, *ε*_*r*_″, *μ*_0_, and *μ*_*r*_″ represent the energy loss per unit volume, electric field intensity, magnetic field intensity, electrical conductivity, frequency, permittivity of free space, dielectric loss permeability of free space, and magnetic loss, respectively. The conductivity, dielectric loss, and magnetic loss are the relevant dielectric parameters for determining the microwave energy absorption of materials. Calcined dolomite has a low loss tangent of 0.000769 at 2.45 GHz and ferrosilicon has a conductivity of 2432 S/m at room temperature. The details of these measurements are presented in the methods section. A briquette consisting of a homogeneous mixture of 80 wt.% calcined dolomite and 20 wt.% ferrosilicon has a low loss tangent of 0.0125 and conductivity of 0.1 S/m, which means that, according to Eq. (2), the briquette cannot be heated well by microwave irradiation. To overcome this, we created a briquette with a microwave resonance structure to confine microwave energy within it (defined as an antenna effect). In other words, the briquettes acted as an antenna with a quarter length of the wavelength of microwaves and/or an integral multiple of the quarter length to capture electromagnetic waves. This was achieved using an antenna, made of ferrosilicon that was placed at the centre of the briquette, with the ferrosilicon particles acting as a continuous conductor. The wavelength (*λ*_*g*_) of microwaves in the dielectric material is given as


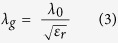


where *λ*_*0*_ and *ε*_*r*_ are the free-space wavelength and relative permittivity of the dielectric material, respectively. As the relative permittivity of the briquette was determined to be between 3.3 and 4.0, the *λ*_*g*_ was estimated to be between 61 and 67 mm at 2.45 GHz. When the length of a briquette is an integral multiple of the quarter wavelength of *λ*_*g*_, it acts as an antenna to capture microwave energy. The briquette was formed using a die made of stainless steel under a 30 MPa pressure applied during the making process, resulting in a briquette with a diameter and height of 13 mm with a deviation of 0.5 mm. Details on these processes are described in the methods section. The five briquettes were stacked to make a tower-shaped assembly with a height of 66 mm, which corresponds to one wavelength.

Three types of briquettes (13 mm in both diameter and height) with different distributions of ferrosilicon in dolomite, (a), (b) and (c), were prepared and their diagonal and cross-sectional views are displayed in Fig. 1(a), (b) and (c). All the briquettes were made of 80 wt.% calcined dolomite and 20 wt.% ferrosilicon particles. The black and white portions in the photographs represent ferrosilicon and dolomite-rich regions, respectively. Ferrosilicon was homogeneously distributed in briquette (a), but concentrated at the centre of briquette (b). Ferrosilicon was inserted into a pillar-shaped hole made at the upper surface of the briquette (the hole was closed at the bottom surface of the briquette) in briquette (c). In terms of microwave frequency, the ferrosilicon particles act as a resistor and inductor, whereas the dolomite particles between the ferrosilicon particles act as a capacitor. These briquettes were then used to make three different antennae structures labelled as (A), (B), and (C)) for use in microwave heating tests.

The experimental setup shown in Fig. 2 was used to confirm the antenna effects while heating and smelting briquettes with various geometries and structures.

Figure 3 shows the temperature profiles observed under microwave irradiation for samples (A), (B), and (C), with a height of 66 mm, which corresponds to one-wavelength of the microwave. The temperature of sample (B) reached 900 °C within 10 min, but sample (C) was not heated at all and is not shown in Fig. 3. The infrared thermometer employed in the present work allows one to measure temperatures above 270 °C, and therefore, only the measured temperature regions are displayed in Fig. 3. The temperature change of sample (A) could not be measured during the first 21 min under microwave irradiation, but then the temperature increased suddenly after 22 min and finally reached 900 °C.

The high frequency characteristics of the three types of antennae structures were also measured, as described in the Supplementary Information, to understand the heating behaviours of these samples. For ideal heating, to capture the microwaves, the antenna should have a high *Q*-value and no parasitic impedance, as compared with a low-*Q* antenna that does not capture the microwaves. Sample (C) had the highest *Q*-value of 983 and no parasitic impedance at its resonant frequency. However, its *Q*-value was too high to capture microwaves, resulting in no rise in its temperature under microwaves generated by a magnetron with a broad-band frequency^15^. However, sample (C) was efficiently heated under microwave irradiation with a narrow-band frequency generated by a solid-state microwave generator (see the Supplementary Information). In contrast, an antenna with low parasitic impedance can be easily matched with microwaves, resulting in little reflection of microwaves. Since sample (B) had a *Q*-value of 691 with parasitic impedance and a capacitance of 1.05 pF at the resonant frequency, this sample could capture microwave energy under microwave irradiation with a broad-band frequency with little matching and was heated well. Sample (A) had an intermediate *Q*-value of 850 and parasitic impedance with a large capacitance of 3.47 pF at its resonant frequency. Sample (A) was heated with a time-delay after microwave irradiation was initiated. Owing to the fluctuations in the microwave frequency generated by a magnetron, accidental impedance matching may have occurred for sample (A) at around 20 min, leading to sudden heating. Additionally, a stirrer fan installed inside the applicator could have induced fluctuations in the distribution of the electromagnetic field, resulting in accidental matching. The lower capacitance for sample (B) compared to that of sample (A) is ascribed to the block of concentrated ferrosilicon particles at the centre.

In addition to this, we also studied two other antenna structures, (D) and (E), shown in Fig. 3. Zirconia beads and glass wool, which have no loss tangent, were used to vary the heights of the samples. Sample (D) was constructed by stacking five pieces of briquette (b) by inserting zirconia beads between each, giving a total height of 78 mm, which corresponded to 1.18 times one wavelength. Sample (E) was constructed by stacking five pieces of briquette (b) and inserting a quartz glass wool separator between two briquettes, giving a total height of 86 mm, corresponding to 1.30 times one wavelength. The temperature of sample (D) started to rise a few minutes after the initiation of microwave irradiation and then reached 900 °C at 15 min. Sample (E) was not heated at all (not shown in Fig. 3). These results prove that the height of the samples should be same as one wavelength of microwave for efficient heating by microwaves.

The electromagnetic (EM) field in the multi-mode applicator was determined by the finite element method (FEM) simulations performed using COMSOL software in order to understand the difference in the temperature behaviours of samples (A) and (B). We employed two models for the simulation: the model of sample (A) was a stack of five briquettes made of an imaginary substance with a complex dielectric constant of 4-0.05*i for a homogenous mixture of 80 wt.% calcined dolomite and 20 wt.% ferrosilicon particles; the model of sample (B) was a tower of five briquettes containing a cylinder block of an imaginary homogeneous substance with the same complex dielectric constant as ferrosilicon at the centre of the same size as the black portion of briquette (b) in Fig. 1. These models were made by simplification of the real samples because a FEM simulation could not be performed with a mesh size smaller than the dolomite and ferrosilicon powder sizes. It was also assumed for sample (B) that the dolomite-rich portion was modelled as an imaginary substance with a complex dielectric constant of 4 -0.05*I, and that the ferrosilicon-rich portion was modelled as an imaginary substance with a conductivity of 2432 S/m in the simulation.

Figure 4 shows the distribution of the electric fields in the applicator containing the samples, in which we see that a strong electric field (standing wave) appeared in the applicator but not in sample (A). On the other hand, a high electric field was accumulated near the briquette of sample (B) due to the confinement of microwave energy in sample (B), with no standing wave in the applicator. These results suggest that the briquettes can capture microwave energy, and be heated to a high temperature, when an antenna structure was constructed in the sample. The temperature of sample (A) finally reached 900 °C in the heating test, even though it should not be heated at all according to the FEM simulation results. This contradiction between the heating test and the simulation might be due to oversimplification of the model in the simulation, i.e., sample (A) consisted of a mixture of the dolomite and ferrosilicon particles, but was modelled as an imaginary substance with homogeneous properties and should be treated as a conductor material with some capacitance at 2.45 GHz frequency in the FEM model simulation.

Mg metal was produced and condensed at the upper part of the inner area of the quartz tube after 80 min of microwave irradiation for sample (B). The operation temperature was 1000 ± 50 °C and a total amount of 1.7 g of Mg metal was obtained, corresponding to a 71% yield, from the raw material, dolomite. A photograph of the obtained Mg metal is shown in Fig. 5. The XRD pattern of the metal confirmed that pure Mg metal was obtained, which demonstrates the viability of using this new hybrid microwave Pidgeon process for producing Mg metal.

The data presented so far establish a new Mg smelting process using microwave irradiation by applying an antenna structure to the starting materials with a low loss tangent. Experiments to investigate the possibility of further reducing the energy used in the microwave Pidgeon processes with an antenna structure in the briquettes were also subsequently carried out.

A large-scale microwave furnace was constructed to obtain over 10 g of Mg metal, in which the idea of the antenna effects was realized in a large-scale reactor compared to the smaller scale reaction used in the former experiment. These specific experiments were designed and conducted to estimate the energy consumption on a large scale. This large-scale experimental setup possessed an applicator under vacuum (600 mm in width, 600 mm in depth, and 900 mm in height), and a microwave output power of up to 6 kW. The new briquettes prepared for the large-scale experiment were designed to have the same structure as briquette (b) in Fig. 1. Six briquettes (11 mm in height and 13 mm in diameter) were stacked to make a tower-shaped assembly with a length of 66 mm, corresponding to one wavelength. Eighteen towers were gathered to form a cylindrical-shaped block. This block had a height ranging between 64 and 68 mm and a diameter ranging between 64 and 68 mm, with a total mass of 265.16 g, and acted as a 3D-antenna. The cylindrical-shaped block was inserted in a quartz tube with an inner diameter of 70 mm. Microwave irradiation was completed after 4 hours, resulting in the temperature of the briquettes exceeding 580 °C, and a green plasma due to Mg vapour was observed. Figure 6(b) also shows a photo of the block, after microwave irradiation was stopped by the reflection of microwaves from the quartz wall covered with deposited Mg metal. Mg metal was successfully obtained at the expected yield of 7.62 g (12%), from which the energy consumption of the newly developed microwave Pidgeon method could be reasonably estimated as 58.6 GJ/t (Mg) (see Supplementary Information). This represents a two-third reduction in energy consumption when compared to the conventional Pidgeon method.

There are many applications for Mg and its alloys as lightweight metals in automotive and battery technologies, and they possess the potential to contribute to future sustainable and low-energy systems. This work demonstrated a new microwave Pidgeon process to curb the high-energy demands and CO_2_ emissions associated with the conventional Pidgeon process. The energy consumed by this new microwave Pidgeon method, based on large-scale batch processing experiments, was estimated to be about three times less than that of the conventional Pidgeon method.

## Methods

**Figure 1:**
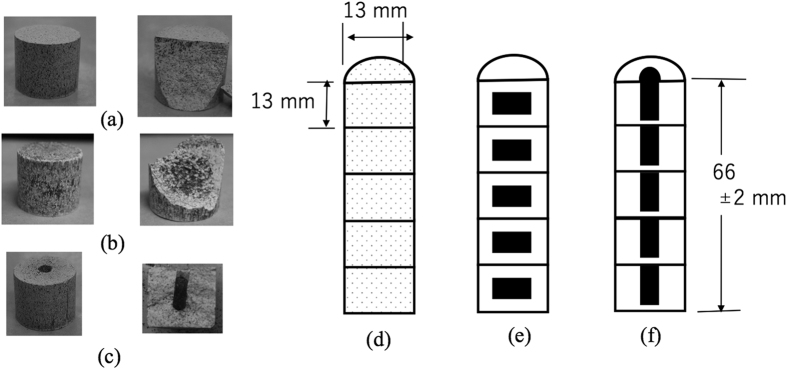
Images of the different types of briquettes and their corresponding antenna structure. The panel on the left shows diagonal and cross-sectional views of briquettes with (**a**) a homogenous mixture, (**b**) a heterogeneous mixture with a concentration of ferrosilicon particles at the centre, and (**c**) a heterogeneous mixture with a rod-shaped column of ferrosilicon particles; the black and white portions in the photographs represent ferrosilicon and dolomite-rich particles, respectively. Schematic of the corresponding antennae: Samples (A), (B), and (C) consist of five briquettes, shown in Fig. 1(a),(b) and (c), respectively, which were stacked in a tower-shaped form.

The complex permittivity of calcined dolomite and ferrosilicon particles was determined using the transmission method (Keysight technologies, N1500A). The conductivity of ferrosilicon particles was determined by the four-point probe method (Loresta-GP MCP-T-610, Mitsubishi Chemical Analytech). The experimental setup for small-scale experiments is shown in Fig. 1 and was constructed by Fuji Electronic Co. Ltd. No specific water cooling system was used for condensing the Mg vapour. The dolomite and ferrosilicon ores were crushed and mixed, then formed into briquettes with diameters and heights of 13 mm. The briquette was formed with a mixture of 80 wt.% calcined dolomite and 20 wt.% ferrosilicon particles using a die made of stainless steel. A pressure of 30 MPa was applied during fabrication, resulting in a briquette with a diameter and height of 13 mm with a deviation of 0.5 mm. The difference between ferrosilicon and dolomite particle sizes determined the distribution of ferrosilicon particles in the briquettes; briquettes with a homogenous mixture were obtained when both had a particle size of 100–200 μm, while those with a heterogeneous mixture were obtained when the particle size of dolomite was 600–700 μm and that of ferrosilicon was 100–200 μm. Three sample types were heated with microwave irradiation with an input power of 800 W in an initial heating test. Each briquette had a non-uniform distribution of ferrosilicon particles to act as an antenna in the briquettes. Following these initial heating tests, sample (A) was selected to be heated by microwave irradiation for the smelting process. Reducing conditions were employed with an average microwave power of 560 W, a temperature of 950–1000 °C (measured using an infrared thermometer, Japan Sensor Corp., FTK9-R), a vacuum pressure of 2 Pa, and a reaction time of 80 min.

**Figure 6:**
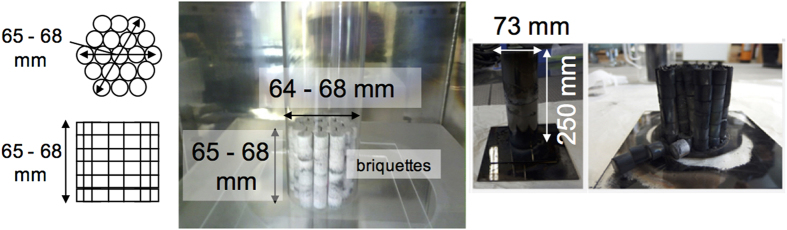
(**a**) Schematic of top and side view of the cylindrical-shaped block obtained using briquette (**b**) in Fig. 1(b) Photograph of dolomite and ferrosilicon briquettes before microwave irradiation, and (**c**) with and without a quartz tube 250 mm in height and 73 mm in diameter after microwave irradiation.

The experimental setup for large-scale experiments is shown in Fig. 6 and was also constructed by Fuji Electronic Co. Ltd. Here, the cavity was evacuated to a pressure of 10^−2^ Pa before the reaction, and was kept at 1.0 to 2.0 Pa during the reaction using diffusion and rotary pumps. The temperature of the surface of the briquettes was measured using an infrared thermometer (Japan Sensor Corp., FTK9-R). The block diagram of the microwave is identical to that of the first experiment. Briquettes with a total mass of 265.16 g were formed into a cylindrical shape with a height and diameter of 66 mm, which corresponded to one wavelength. All free standing briquettes were placed on a quartz plate and surrounded by a quartz tube. The temperature of the briquettes was also measured as a function of microwave irradiation time (Fig. 6) to obtain the efficiency of the transfer of microwave irradiation power to the material. The temperature of the briquettes increased to over 550 °C in 5 min, and a green plasma from the Mg vapour was observed. Microwave irradiation was applied to the material until the reaction ended after 250 min and the green plasma ceased. All experiments were safely carried out, because the reaction took place under vacuum and the reaction temperature did not exceed 1200 °C, which was lower than the melting point of quartz.

## Additional Information

**How to cite this article**: Wada, Y. *et al*. Smelting Magnesium Metal using a Microwave Pidgeon Method. *Sci. Rep.* **7**, 46512; doi: 10.1038/srep46512 (2017).

**Publisher's note:** Springer Nature remains neutral with regard to jurisdictional claims in published maps and institutional affiliations.

## Supplementary Material

Supplementary Information

## Figures and Tables

**Figure 2:**
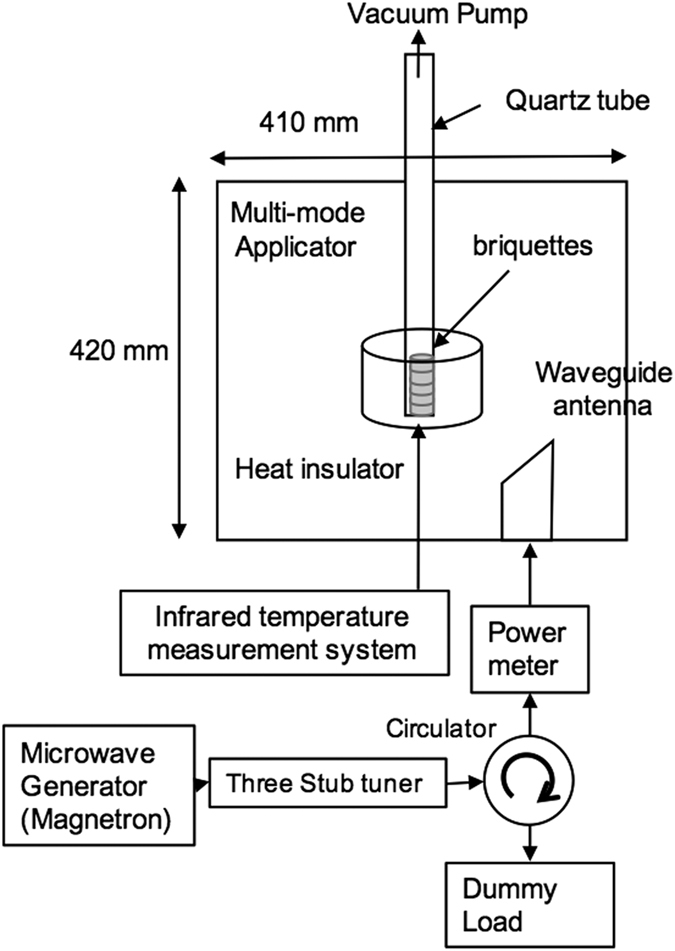
Experimental setup for the microwave Pidgeon method comprising a multi-mode applicator (410 mm in width, 410 mm in depth, and 420 mm in height), a stirrer fan, and a magnetron as a microwave source. Briquettes were located in the cavity center in a quartz tube evacuated to 1 Pa.

**Figure 3:**
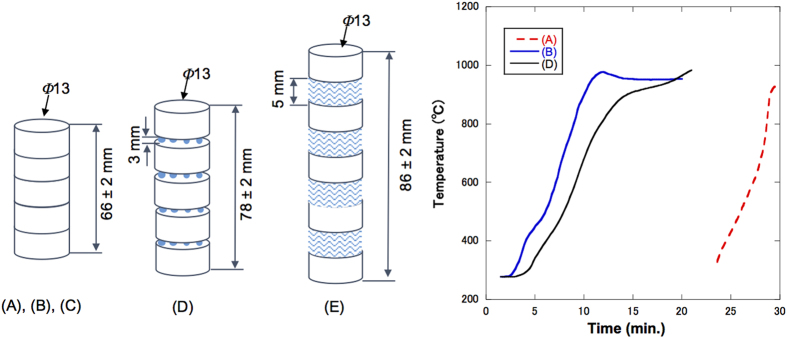
Schematic of the different antenna types and a plot of the measured temperatures as a function of microwave irradiation time. Samples (**A**), (**B**), and (**C**) contain a stack of five briquettes, using briquettes (a), (b), and (c), respectively (height of one-wavelength (66 mm). Sample (**D**) consists of five briquettes (b), stacked with zirconia beads between each two briquettes to a total height of 78 mm. Sample (**E**) contains five briquettes (b), stacked with four quartz glass wool separators to a total height of 86 mm. The measured temperature for each antenna type as a function of microwave irradiation time is shown on the right.

**Figure 4:**
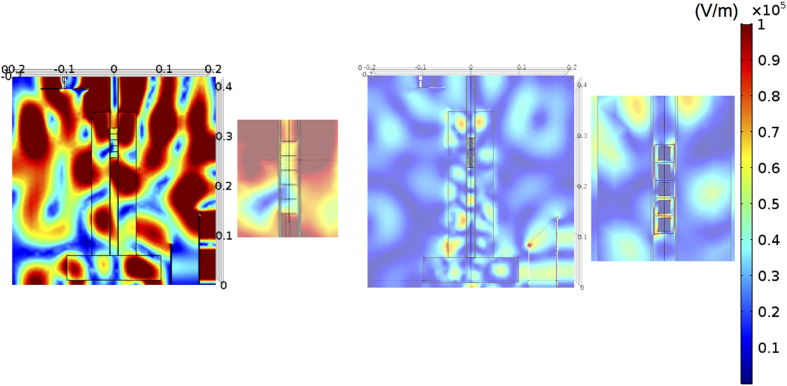
Finite element method simulations of applicator and briquettes. (**a**) the model of sample. (A), and (**b**) the model of sample (B).

**Figure 5:**
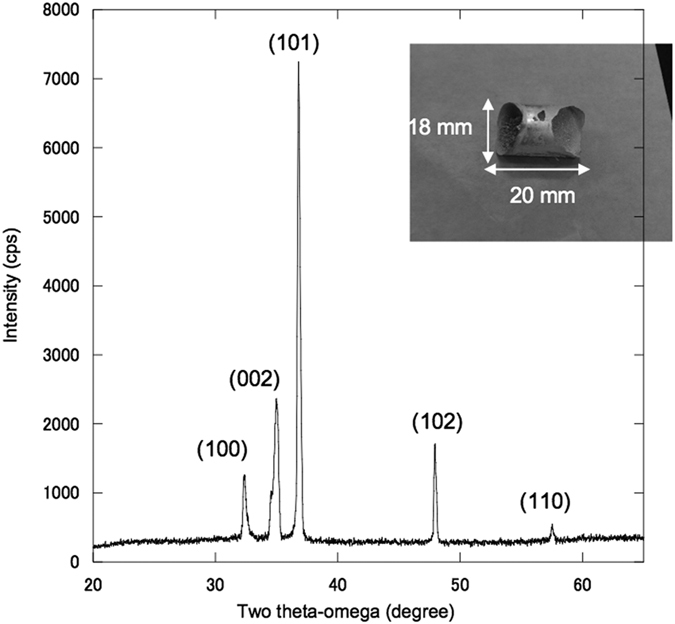
X-ray diffraction pattern of the Mg that condensed on the quartz tube. All peaks were identified as Mg face numbers. The inset shows a photograph of the metal sample having a diameter of 18 mm, maximum length of 20 mm, and thickness of 1 mm.
