# Supplementary material for: Smelting Magnesium Metal using a Microwave Pidgeon Method
Source: Sci Rep. 2017 Apr 12;7:46512. doi: 10.1038/srep46512 (PMC5388895; doi:10.1038/srep46512)
Supplement: Supplementary Information [file srep46512-s1.docx]

**Smelting Magnesium Metal using a Microwave Pidgeon Method**

Authors: Yuji Wada^1*^, Satoshi Fujii^1,#,*^, Eiichi Suzuki^1^, Masato M. Maitani^1##^, Shuntaro Tsubaki^1^, Satoshi Chonan^2^, Miho Fukui^2^, Naomi Inazu^1^

Affiliation: ^1^Graduate School of Science and Engineering, Tokyo Institute of Technology, 2-12-1Ookyama, Meguro-ku, Tokyo, 152-8550 Japan

^2^Oricon Energy Inc., 6-8-10 Roppongi, Minato-ku, Tokyo, 106-0032 Japan

# Present address: Department of Information and Communication System Engineering, National Institute of Technology, Okinawa College, 980 Henoko, Nago-shi, Okinawa, 905-2192 Japan

# # Present address: Research Center for Advanced Science and Technology, The University of Tokyo, 4-6-1, Komaba, Meguro-ku, Tokyo, 153-8904 Japan.

*Correspondence to: E-mail: yuji-w@apc.titech.ac.jp (Y. W.), fujii.s.ap@m.titech.ac.jp (S. F.)

**Supplementary information**

*Measurement of frequency characteristics of samples (A), (B), and (C)*

The frequency characteristics of samples (A), (B), and (C) were measured in a single-mode-cavity of the TE103 using a network analyzer (Rode & Suwaltue, ZND). Extended Data Figure 1 shows the scattering parameter, *S11*, and impedance, *z11*, of the five briquettes in the TE103 waveguide cavity. Sample (C) had an *S11* amplitude of over -30 dB with little parasitic impedance and high *Q*-value of 983 at its resonant frequency, and therefore acted as a narrow-band antenna. This suggests that 99.9 % of the microwave power was absorbed by the briquettes. Sample (A) had an *S11* amplitude of about -15 dB and some parasitic impedance, with a capacitance of 3.47 pF and *Q-*value of 850 at its resonant frequency. Sample (B), however, had an *S11* amplitude of -6 dB and parasitic impedance, with a capacitance of 1.05 pF and *Q-*value of 691 at the resonant frequency due to the added inductance provided by the block of ferrosilicon, which acted as a broad-band antenna. As a result, only 75 % of the microwave power was absorbed over a bandwidth of about 5 MHz. In the small-scale experiment, briquettes were used as a broad-band antenna, because the magnetron microwave source generated a spectrum that was wider than the range of the narrow-band antenna. In addition, the centre frequency of the magnetron was not stable to within 5–10 MHz.^1^ This makes it vital that the frequency be matched to the impedance in order to reduce the reflection power.


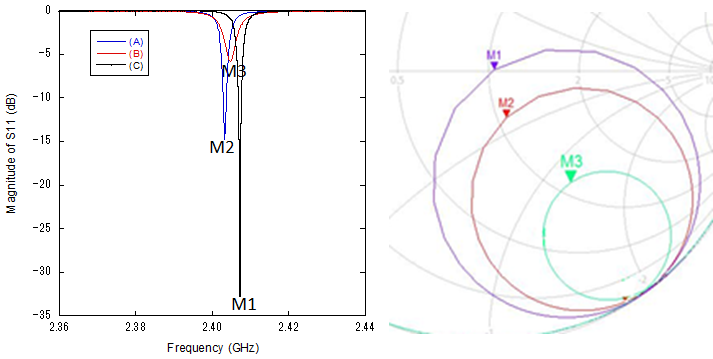


**Figure 1.** *S11* measurement results for briquettes with antenna structures; Samples (A), (B), and (C), (a) S11 magnitude, (b) *Z11* Smith chart, M1, M2 and M3 show the resonance frequency of each briquette.

*Heating test using TE103 cavity and a solid-state microwave generator*

Samples (A), (B), and (C) were heated in the TE103 single-mode cavity under microwave irradiation using a solid-state microwave generator with an output power of 150 W. Extended Data Figure 2 shows the measured temperature of samples (B) and (C), which is described in Figure 1, and the microwave power as a function of the reaction time. Sample (C) reached a temperature of 500 °C faster than sample (B). This result is quite reasonable based on the measurement of high-frequency characteristics of samples (B) and (C), using a solid-state microwave generator with stable and narrow-band frequency. Five consecutive briquettes stacked to a height of one wavelength (66 mm) with a rod-shaped antenna have a high *Q-*value and no parasitic impedance, compared to briquettes having a heterogeneous mixture with ferrosilicon particles concentrated at the centre.


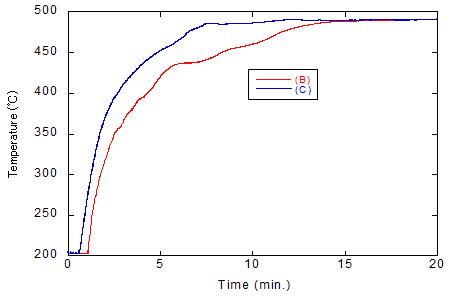


**Figure 2.** The measured temperature as a function of microwave irradiation time. (a) Briquette types: sample (B) five briquettes piled up to a height of one wavelength (66 mm) with a heterogeneous mixture with a concentration of ferrosilicon particles at the centre, and sample (C) five briquettes piled up to a height of one wavelength (66 mm) with a heterogeneous mixture with a rod-shaped column of ferrosilicon particles.

*Estimation of energy consumption using the microwave Pidgeon method*

The energy consumption using the microwave Pidgeon method was estimated from the temperature increase in the microwave chamber during the large-scale batch process. Only the energy consumed during heating of the dolomite-ferrosilicon pellets was estimated based on the measured temperature increase (Extended Data Figure 2) and the heat capacity of the pellet materials (1.15 kJ/kg for dolomite^2^ and 0.80 kJ/kg for ferrosilicon^3^). Extended Data Figure 3 shows the temperature increase of a cylindrical-shaped block (265 g), as observed by an infrared radiation thermometer. The input and returned microwave power are also displayed in the same plot. The effective microwave power directly applied to the target pellets was estimated using Eq. (2). The heat energy transferred to the target pellets was also calculated using Eq. (3). The efficiency of energy conversion from the applied microwaves to thermal energy, which heated the target block, was subsequently defined by the ratio E_Therm_/E_MW_. From the experimental results, the calculated energy conversion efficiency from microwave to heat energy was approximately *η_MWheat_* = 0.37. The realistic energy consumption was evaluated as the electric energy for the microwave Pidgeon method. This energy consumption was calculated by considering the conversion efficiency from the electric source to the microwaves generated by a typical magnetron source (*η_MWgen_* = 0.7), the energy conversion efficiency from microwaves to the heating of the target block (*η_MWheat_* = 0.37), and the 75 % yield of the Pidgeon method (*η_Mg_* = 0.75) from dolomite (0.35 wt.% Mg) when the target block was heated to 1,100 °C. As the chemical reduction of MgO by Si is an endothermic reaction, this heat energy was considered alongside the energy supplied by microwave heating and calculated using Eq. (4). The energy consumed by the microwave Pidgeon method calculated using Eq. (4) was *E_Mgt_* = 58.6 GJ/t (Mg), which is 31.4 % of that used by the conventional Pigeon method using coal-based energy.

$E_{MW}=\int_{t_{0}}^{t_{1}} (P_{FWD}-P_{REV})dt$ $E_{\mathrm{MW}}=\int_{t_{0}}^{t_{1}} (P_{\mathrm{FWD}}-P_{\mathrm{REV}})dt$ $E_{\mathrm{MW}}=\int_{t_{0}}^{t_{1}} (P_{\mathrm{FWD}}-P_{\mathrm{REV}})dt$ (2)

where *t_0_* and *t_1_* are the initial and final times when the sample temperature was measured, respectively, and *P_FWD_* and *P_REV_* are the forward and reverse microwave power, respectively.

$E_{Therm}=\int_{T_{0}}^{T_{1}} (M_{dol}C_{p dol}-{M_{FS}C}_{p FS})dT$ (3)

where *T_0_* and *T_1_* are the measured initial and final pellet temperatures during the heating experiment, respectively, as indicated in Extended Data Figure 3, and *M* and *C_p_* are the weight and specific heat capacity, respectively, of dolomite and ferrosilicon. The values of *M_dol_* for dolomite (0.35 wt.% Mg) and *M_FS_* for ferrosilicon for 1 t of Mg production were 3809.52 and 1039.26 kg, respectively, assuming a production yield of 75 %.

$$E_{Total}=\frac{{{(E}_{Therm}+E_{react}})}{\left( \eta_{MWgen}\times\eta_{MWheat}\times\eta_{Mg} \right)}$$

$=\left[ \left\{ \int_{25℃}^{1100℃} (M_{dol}C_{p dol}+{M_{FS}C}_{p FS})dT \right\}+\left\{ \Delta H-\Delta(TS) \right\}_{T=1100℃} \right]/\left( \eta_{MWgen}\times\eta_{MWheat}\times\eta_{Mg} \right)$ (4)

$=\frac{\left[ \left\{ \int_{25℃}^{1100℃} (3809.52[kg] \times1.15[kJ/kg\cdot K ]M_{dol}C_{p dol}-\mp1039.26[kg] \times0.8[kJ/kg\cdot K ]{M_{FS}C}_{p FS})dT \right\}+9.63\left\{ \Delta H-\Delta(TS) \right\}_{T=1100℃} \right]}{\left( 0.7\times0.37\times0.75 \right)}$ (5)

where *E_reac_*_t_ is the reaction energy, which is defined by the change in Gibbs free energy.


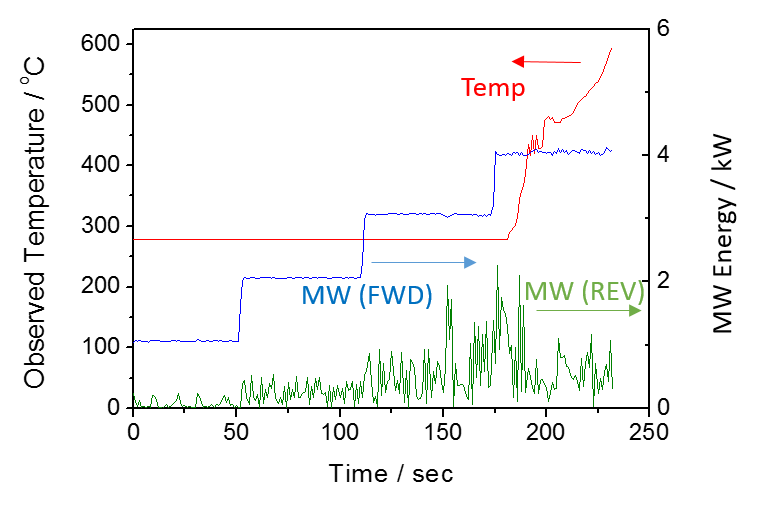


**Figure 3.** Temperature profile of a cylindrical-shaped block in a large-scale cavity as a function of microwave irradiation time.

**References**

[1] Fujii, S., et al. Chemical reaction under highly precise microwave irradiation. *J. Microwave Power EE*. **48,** 89-103(2014)

**[**2**]** Kenneth M., et al. High-temperature heat capacities and derived thermodynamic properties of anthophyllite, diopside, dolomite, enstatite, bronzite, talc, tremolite and wollastonite. *American Mineralogist*, **70**, 261-271(1985)

**[**3**]** Elkem LC FeSi 75 Low Carbon Ferrosilicon, Materials Database, http://www.matweb.com, accessed on 3/3/2017.
